# Age-stratified anti-Müllerian hormone (AMH) nomogram: a comprehensive cohort study including 22.920 women

**DOI:** 10.3389/fendo.2025.1612194

**Published:** 2025-06-20

**Authors:** Kiper Aslan, Isil Kasapoglu, Bahadir Kosan, Aylin Tunali, Ilayda Tellioglu, Gurkan Uncu

**Affiliations:** Department of Obstetrics and Gynecology, Faculty of Medicine, Bursa Uludag University, Bursa, Türkiye

**Keywords:** AMH, ovarian reserve, age stratification, diminished ovarian reserve, nomogram, infertility, endometriosis

## Abstract

**Background:**

Infertility rates have been rising globally, necessitating accurate assessment tools for ovarian reserve. Anti-Müllerian hormone (AMH) is a key biomarker for evaluating ovarian reserve, yet age-stratified reference data remain limited. Establishing an AMH nomogram could enhance fertility counseling and treatment planning.

**Objective:**

To develop an age-stratified AMH nomogram to improve the understanding of ovarian reserve across reproductive ages and assist in comparing individual AMH values with age-specific thresholds, aiding in the baseline infertility work-up

**Methods:**

This retrospective cohort study analyzed AMH test results from a tertiary university hospital’s electronic database between April 2015 and June 2024. Data were collected from various departments, excluding women younger than 18 or older than 45 years. Median AMH levels and interquartile ranges were calculated for each age group. The prevalence of diminished ovarian reserve (DOR), defined as AMH <1.2 ng/mL, was determined. Statistical analyses, including correlation testing and subgroup comparisons across different clinical settings, were performed using SPSS version 22 (IBM Corp., Armonk, NY, USA).

**Results:**

A total of 22,920 AMH results were analyzed after excluding patients outside the 18–45 age range and those with incomplete data. More than half of the AMH tests were from women aged 24–33 years. The results demonstrated a significant negative correlation between age and AMH levels, with a median AMH value dropping below 1.2 ng/mL by age 36. The prevalence of DOR increased from 15.9% at age 18 to 96% at age 45. Additionally, women from the Endometriosis Unit had significantly lower AMH levels (median 1.6 ng/mL) compared to other departments (median 2.03 ng/mL). The age-stratified AMH distribution remained consistent even when patients from ART (Assisted Reproductive Technology) centers, REI (Reproductive Endocrinology and Infertility), and the Endometriosis Unit were excluded.

**Conclusion:**

This study provides an age-stratified AMH nomogram that can serve as a valuable tool for clinicians to assess ovarian reserve more accurately. The sharp decline in AMH levels, particularly after age 36, emphasizes the need for timely fertility evaluations and interventions, particularly in populations at risk for diminished ovarian reserve, such as those with endometriosis.

## Background

The global infertility rate has been rising dramatically over the past few decades (1). Various factors contribute to infertility, including male factor, tubal factor, unexplained infertility, endometriosis, advanced age, and diminished ovarian reserve (2). Regardless of the underlying cause, a comprehensive infertility work-up for all couples remains essential. This evaluation includes a detailed history from both partners, physical examination, semen analysis, tubal assessment, ultrasonographic evaluation of the female pelvic anatomy, and ovarian reserve testing (3). Assessing ovarian reserve is a critical step in determining the appropriate treatment plan.

urrent practice primarily evaluates ovarian reserve using two key parameters: anti-Müllerian hormone (AMH) levels and antral follicle count (AFC) (4, 5). AMH is a glycoprotein hormone secreted by the granulosa cells of preantral and small antral follicles in the ovaries. It serves as a reliable endocrine marker of the remaining follicular pool, reflecting a woman’s reproductive potential. The antral follicle count is a useful, easy-to-perform, and cost-effective method, though it is ultrasound- and operator-dependent (6, 7). Additionally, conditions such as endometriomas may interfere with accurate AFC measurement. In such cases, AMH levels become particularly important for assessing ovarian reserve (8).

The existing literature provides substantial data on AFC and AMH concerning infertility evaluation, predicting controlled ovarian hyperstimulation (COH) outcomes, and forecasting cumulative live birth rates (9, 10). However, an equally important factor that influences all assisted reproductive technology (ART) outcomes is the woman’s age (11). Unfortunately, the literature lacks clarity regarding age-stratified ovarian reserve parameters.

A recent study presents age-stratified AFC outcomes to guide patients, offering AFC results by age, which can help in comparing ovarian reserve against age-matched thresholds (12). Similarly, another study from China provides quartiles and median AMH levels by age for a healthy population (13). To contribute to this growing body of knowledge, we aim to establish an AMH nomogram. This will make it easier to inform patients about their ovarian reserve, comparing their results with those of women in the same age group during their baseline infertility work-up.

## Methods

### Study design and ethical approval

This retrospective cohort study was conducted at a tertiary university hospital, utilizing an electronic database. Ethics approval for the study was obtained from the Bursa Uludag University Clinical Trials Ethical Committee with the number 2024-19/5.

### Data collection and patient enrollment

The hospital’s electronic database was screened for AMH results collected between April 2015 and June 2024. AMH values from all departments, including Obstetrics and Gynecology (Ob&Gyn), were included. Women younger than 18 years or older than 45 years at the time of testing were excluded. Data collected included patient age at the time of testing and the department where the test was ordered. Records with incomplete or insufficient data were excluded from the analysis.

### AMH analysis

The hospital’s standard protocol for AMH testing involved morning blood sample collection, typically while patients were fasting, by trained laboratory staff. The samples were processed under the appropriate conditions for AMH assays. Over the 10-year study period, the AMH levels were measured using the “Beckman Coulter Access II” enzymatic immunoassay, and results were recorded in ng/mL.

### Statistical analysis

AMH levels were stratified by the patient’s age, ranging from 18 to 45 years. Median AMH levels were calculated for each age group, along with the 25th and 75th percentiles. According to the Poseidon Classification (14), diminished ovarian reserve (DOR) was defined as an AMH level below 1.2 ng/mL, and the percentage of women with DOR was calculated for each age group. To minimize bias, a separate analysis was performed, excluding patients who had been seen in infertility clinics. All statistical analyses were performed using SPSS software version 22.0 (IBM Corp., Armonk, NY, USA).

## Results

**Figure 1:**
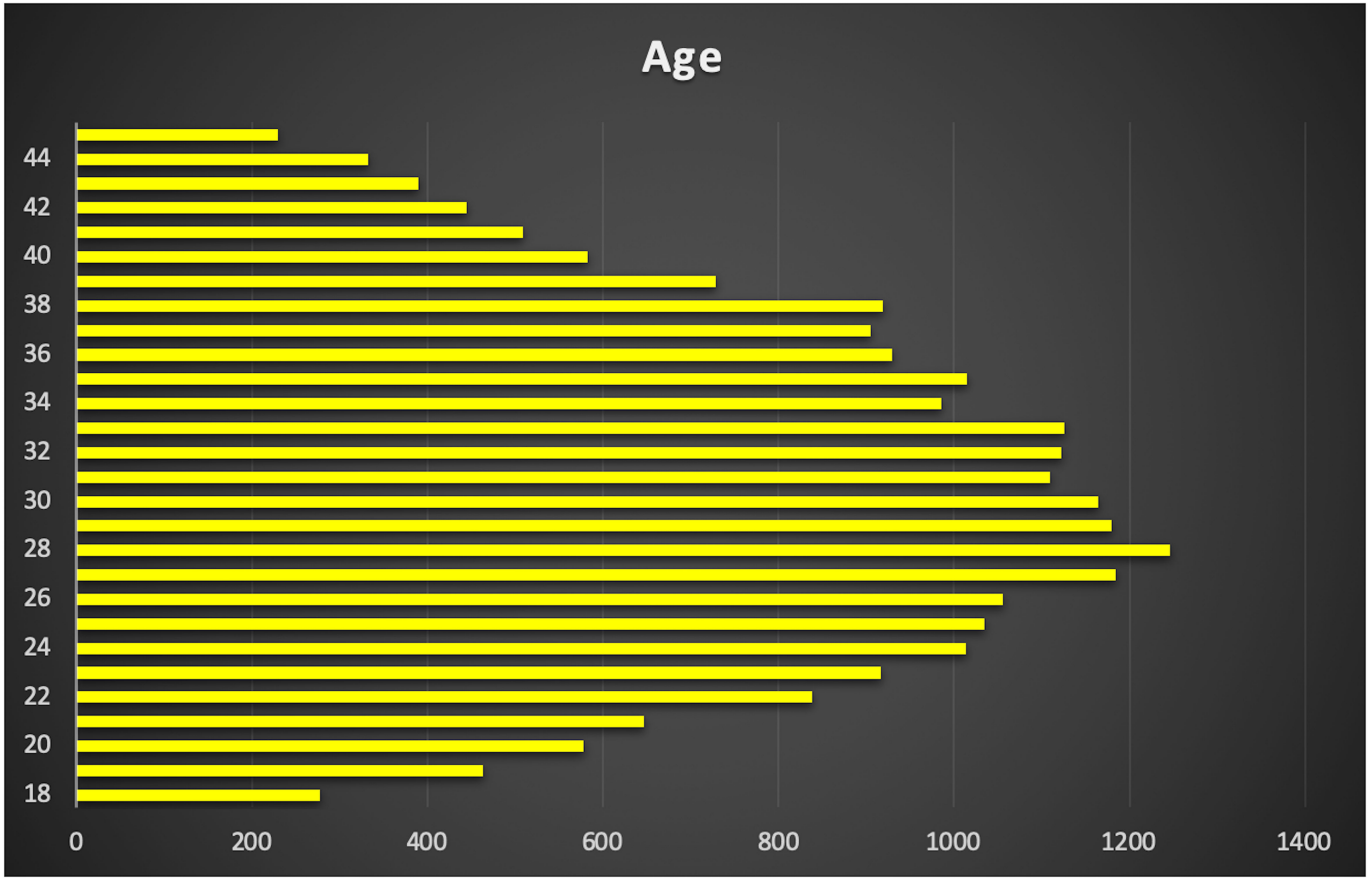
Distribution of AMH & women age.

**Figure 2:**
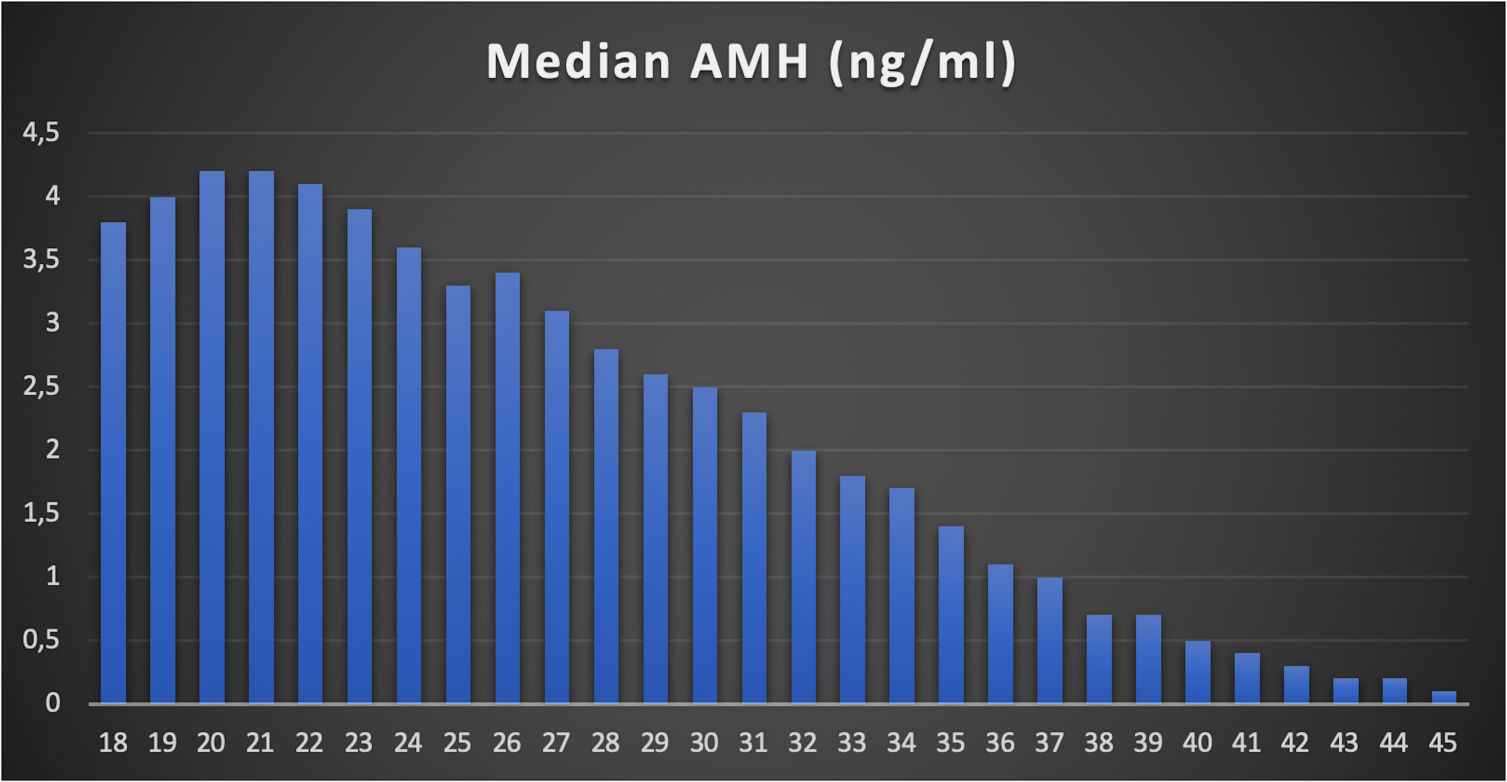
Median AMH value in each age (ng/ml).

**Figure 3:**
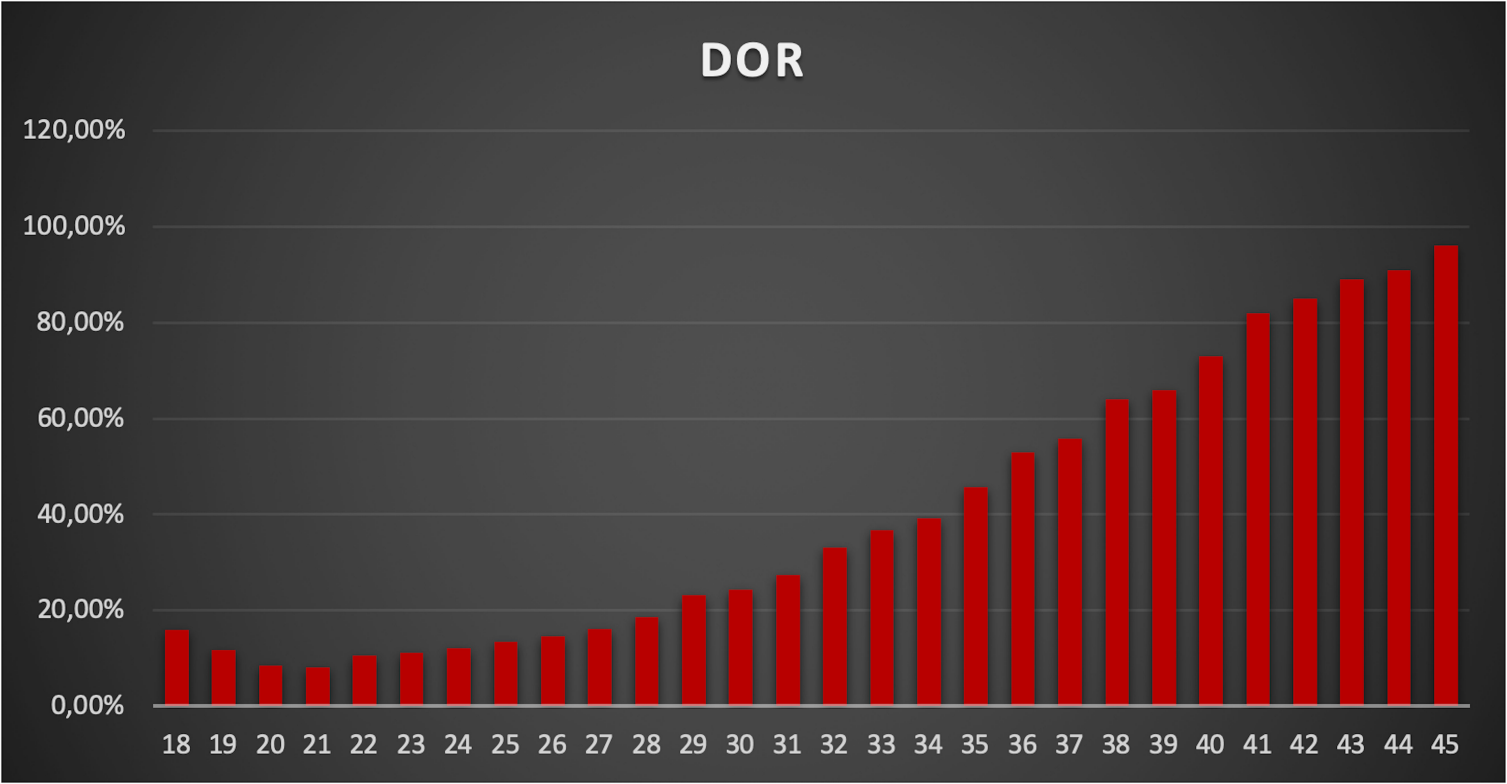
Presence of diminished ovarian reserve in each age.

A total of 24,587 AMH results were retrieved from the hospital’s electronic database spanning from April 2015 to June 2024. After excluding patients below 18 and above 45 years old, along with those with incomplete data, 22,920 AMH results were included in the final analysis (**Table 1**). More than half of these results were from women aged between 24 and 33 years (**Figure 1**). Correlation analysis revealed a significant inverse relationship between AMH levels and age; as age increased, AMH levels progressively declined (**Figure 2**). By the age of 36, the median AMH values fell below 1.2 ng/ml. The prevalence of diminished ovarian reserve (DOR) was 15.9% at age 18, escalating to almost 96% by the age of 45 (**Table 1**, **Figure 3**).

**Table 1:** Age stratified AMH values and ratio of diminished ovarian reserve.

Age	Number of Pts N = 22920	Median AMH (ng/ml)	25%	75%	AMH <1.2 (ng/ml)
18	277	3.8	1.9	7	15.90%
19	463	4	2.3	6.8	11.70%
20	578	4.2	2.5	6.7	8.50%
21	647	4.2	2.6	6.8	8.20%
22	838	4.1	2.2	6.4	10.50%
23	916	3.9	2.1	6.3	11.20%
24	1013	3.6	2	6.1	12.20%
25	1035	3.3	1.9	5.7	13.50%
26	1055	3.4	1.9	6	14.60%
27	1184	3.1	1.7	5.3	16.20%
28	1246	2.8	1.5	4.9	18.60%
29	1180	2.6	1.3	4.6	23.20%
30	1164	2.5	1.2	4.3	24.30%
31	1109	2.3	1.1	3.9	27.30%
32	1122	2	0.9	3.8	33.20%
33	1126	1.8	0.8	3.3	36.70%
34	985	1.7	0.7	3.3	39.30%
35	1015	1.4	0.5	2.9	45.70%
36	929	1.1	0.4	2.3	52.90%
37	905	1	0.3	2.3	55.80%
38	919	0.7	0.2	1.7	64%
39	728	0.7	0.2	1.6	66%
40	582	0.5	0.2	1.3	73%
41	509	0.4	0.1	0.9	82%
42	444	0.3	0.1	0.8	85%
43	390	0.2	0.1	0.6	89%
44	332	0.2	0.1	0.5	91%
45	229	0.1	0.05	0.3	96%

**Figure 4:**
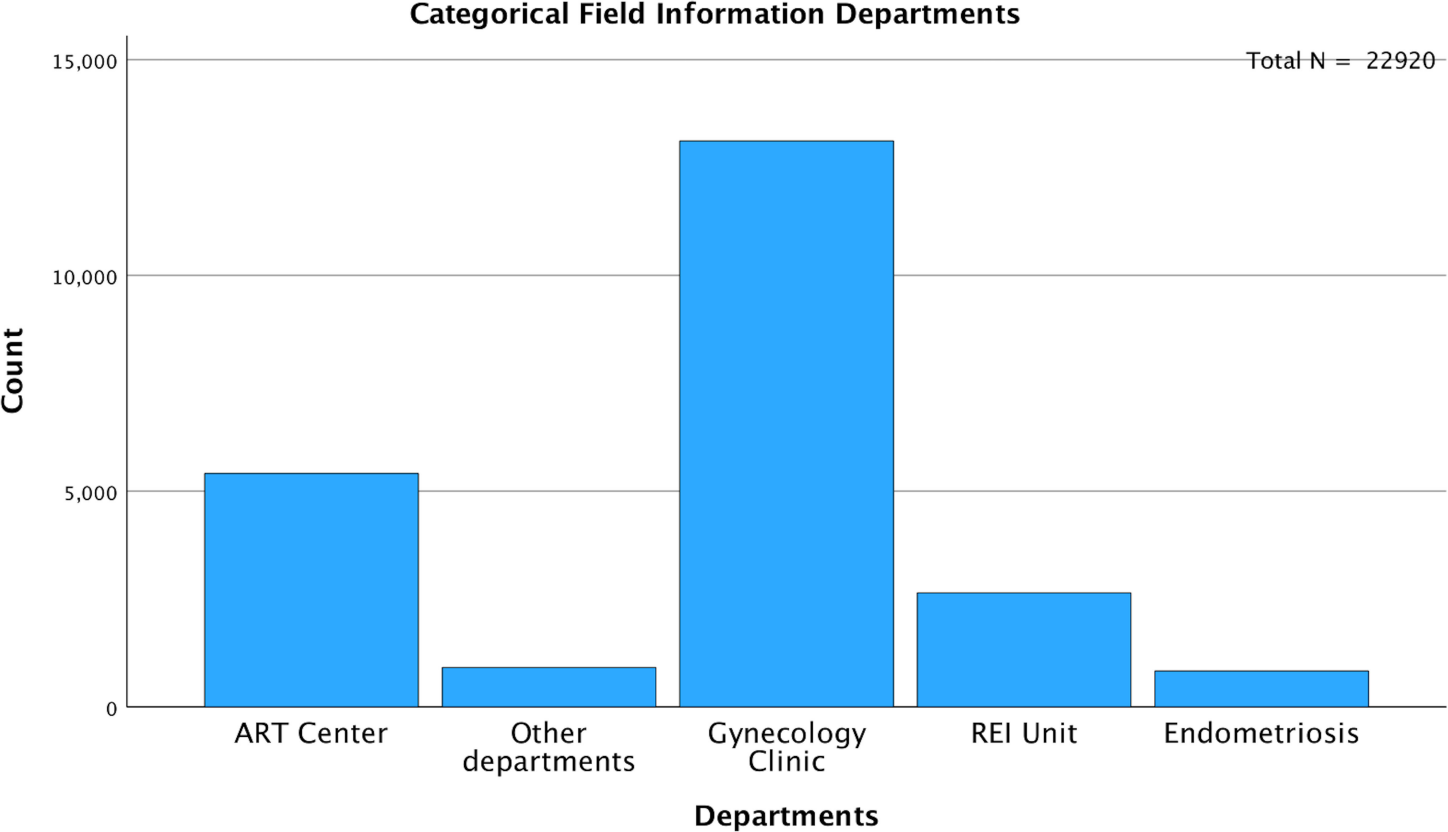
Distribution of AMH Samples.

Among the entire cohort, 5,413 (23.6%) samples were gathered from the ART Center, 2,644 (11.5%) from the REI unit, and 834 (3.6%) from the Endometriosis Center. The remaining 14,029 (61.2%) samples were from departments including gynecology, endocrinology, dermatology, and internal medicine (**Figure 4**). To minimize bias, an additional analysis was conducted after excluding patients from the ART Center, REI unit, and Endometriosis Center (N=14,029). The age-stratified median AMH values in the excluded population (Gynecology Clinic: N=13,114, Other Departments: N=915) were comparable to those from the ART Center (N=5,413), REI unit (N=2,644), and Endometriosis Center (N=834) (**Table 2**).

**Table 2:** Age stratified AMH values and presence of DOR (ART Center, REI and Endometriosis Unit Excluded).

Age	Number of Pts N = 14029	Median AMH (ng/ml)	25%	75%	AMH <1.2 (ng/ml)
18	261	3.8	1.9	7	16.1%
19	428	4.2	2.4	6.9	11.9%
20	505	4.3	2.5	6.6	7.9%
21	544	4.4	2.6	6.9	8.5%
22	666	4.0	2.2	6.7	10.8%
23	664	4.1	2.2	6.6	11%
24	705	3.7	2.2	6.2	10.6%
25	653	3.4	2.0	6.0	11.8%
26	642	3.5	2.0	5.9	14%
27	667	3.1	1.6	5.3	18.1%
28	657	2.8	1.5	5.1	20.2%
29	666	2.6	1.4	4.7	21.8%
30	596	2.6	1.1	4.4	26.3%
31	587	2.4	1.2	4	25.7%
32	566	1.9	0.8	3.7	36.2%
33	566	1.9	0.7	3.5	39.4%
34	531	1.6	0.7	3.4	44%
35	525	1.5	0.6	3.1	54.6%
36	478	1.1	0.3	2.3	56.4%
37	463	1	0.3	2.1	67.3%
38	477	0.6	0.2	1.5	65.6%
39	398	0.7	0.1	1.6	73.8%
40	389	0.4	0.1	1.2	81.9%
41	354	0.3	0.1	0.9	85.7%
42	322	0.3	0.1	0.8	91%
43	288	0.2	0.1	0.6	90.4%
44	251	0.2	0.1	0.5	95.6%
45	180	0.1	0.05	0.3	96%

9.

**Figure 5:**
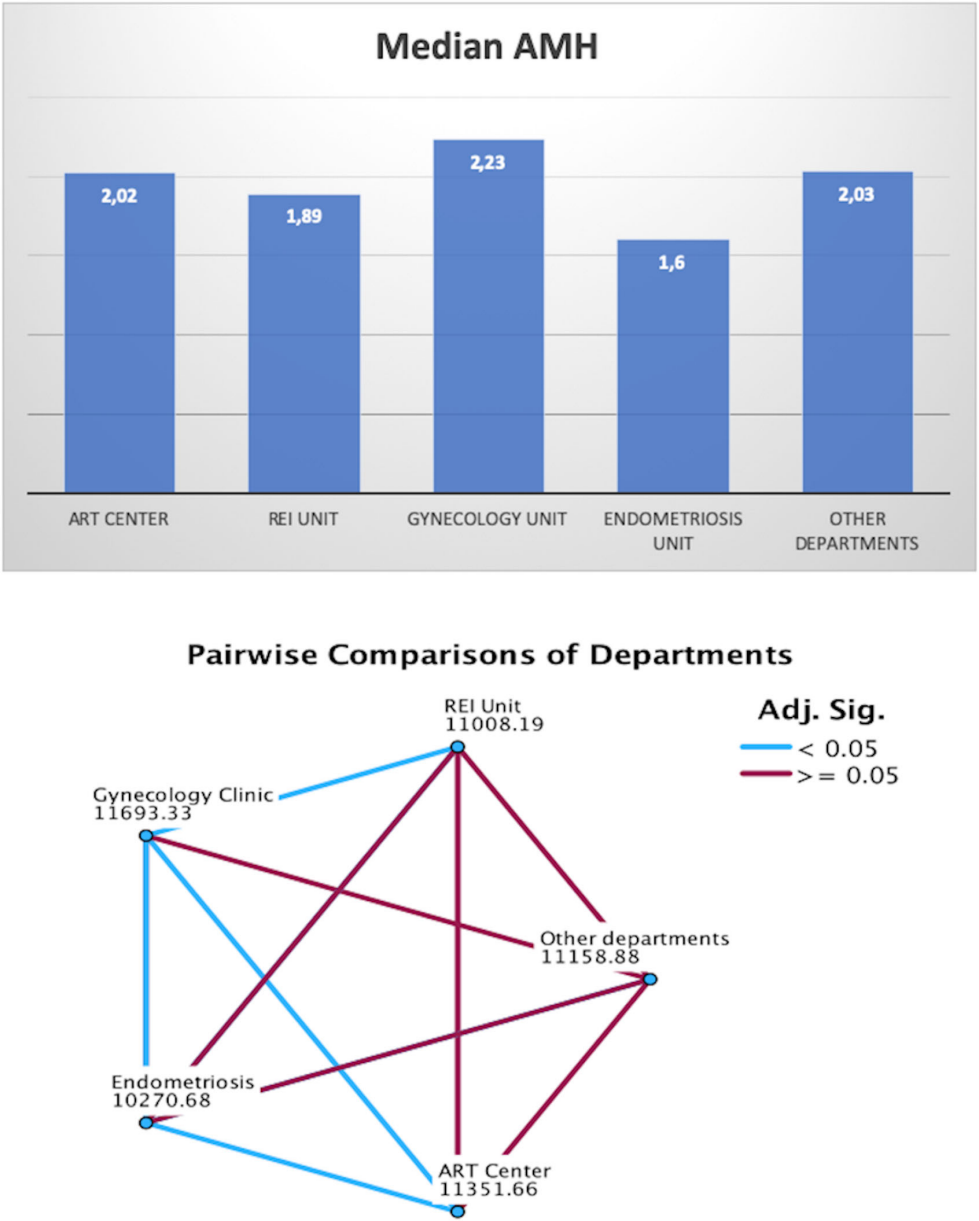
Presence of diminished ovarian reserve in each department & pairwise comparisons.

**Figure 6:**
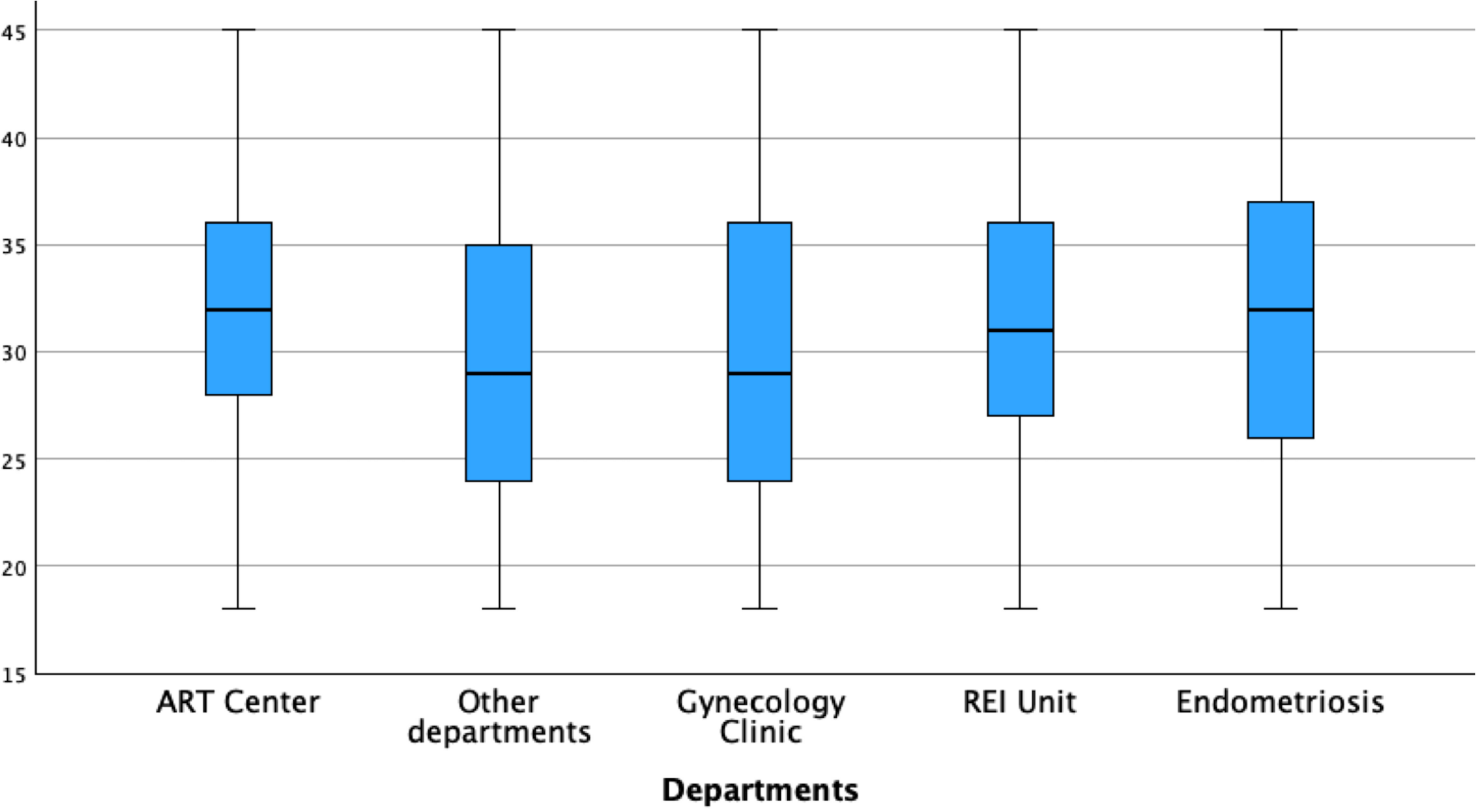
Women age & departments.

When stratifying AMH values by department, the Endometriosis Center exhibited the lowest median AMH levels (1.6 ng/ml), followed by the REI Unit (1.89 ng/ml), ART Center (2.02 ng/ml), Other Departments (2.03 ng/ml), and Gynecology Clinic (2.23 ng/ml) (**Figure 5**). Pairwise comparisons of AMH values between departments were illustrated in **Figure 5**. The average age of women was slightly higher in the Endometriosis Center (31.9 ± 6.8 years) compared to other departments (REI Unit: 31.3 ± 5.8 years, Gynecology Clinic: 30.1 ± 7.1 years, ART Center: 31.6 ± 5.2 years, Other Departments: 29.8 ± 7.3 years) (**Figure 6**).

## Discussion

The results of this study provide further insights into the relationship between anti-Müllerian hormone (AMH) levels and age in a diverse population. AMH is a well-established marker of ovarian reserve, and its use in clinical practice continues to expand as we gain a deeper understanding of its role in assessing fertility potential (15). In this study, we have stratified AMH values by age and demonstrated significant age-related decline in AMH levels, supporting existing evidence in the literature (16–18). Specifically, by the age of 36, the median AMH value drops below 1.2 ng/mL, a commonly used threshold for diminished ovarian reserve (DOR) (19, 20). The prevalence of DOR rises steeply with age, from 15.9% at age 18 to nearly 96% by age 45, illustrating the rapid decline in ovarian reserve, particularly after the mid-30s.

The stratified data further reveal that the median AMH levels remain relatively stable during the early reproductive years, peaking around age 20 and then gradually declining thereafter. By age 30, a noticeable shift occurs, with AMH levels dropping from a median of 2.5 ng/mL at age 30 to 1.4 ng/mL by age 35. These findings are in line with prior studies that demonstrate a sharp decrease in AMH during the late 30s, reflecting the accelerated loss of ovarian follicles during this period (21).

Furthermore, the study compared AMH values across different clinical departments, shedding light on the impact of specific conditions like endometriosis on ovarian reserve. Women treated in the Endometriosis Unit had the lowest median AMH levels, which were significantly lower than those in the Gynecology Clinic, ART Center, or REI Unit. This observation is consistent with the hypothesis that endometriosis, particularly when associated with ovarian involvement (endometriomas), may accelerate ovarian reserve depletion through inflammatory or mechanical damage to the ovarian tissue (22–24). These findings underline the importance of early fertility assessment in women with endometriosis to offer timely interventions, such as fertility preservation.

The exclusion of patients from infertility-specific clinics (ART Center, REI Unit, and Endometriosis Center) revealed similar age-stratified AMH patterns in the general population, reinforcing the general applicability of the AMH nomogram. The similarity in results across these subpopulations suggests that AMH testing can reliably assess ovarian reserve regardless of the clinical setting, though certain conditions, like endometriosis, warrant special consideration.

These age-stratified AMH data provide clinicians with a valuable tool to counsel patients about their reproductive potential. The ability to compare a patient’s AMH level with age-specific reference ranges allows for more personalized fertility assessments. For example, a 30-year-old woman with an AMH of 2.5 ng/mL may be reassured that her ovarian reserve is within the expected range for her age, while a woman of the same age with an AMH 1.4 ng/mL could be counseled about the potential for diminished ovarian reserve and options for fertility preservation or more immediate intervention.

The clinical implications of these findings are profound. AMH testing can help guide decisions about fertility treatment, timing of family planning, and the need for interventions such as oocyte cryopreservation. Moreover, for patients undergoing assisted reproductive technologies (ART), age-stratified AMH data can assist in predicting ovarian response to stimulation and tailoring treatment protocols.

One notable strength of our study is the large sample size, which includes 22,920 AMH results collected over a nearly decade-long period. This has allowed us to provide robust age-stratified AMH data, contributing valuable information to existing nomograms. Our study population is unique in its inclusion of patients from a wide range of clinical settings, including infertility clinics, gynecology, endocrinology, and even departments not primarily focused on reproductive health. This diversity enhances the generalizability of our findings.

Despite the strengths of this study, including the large sample size and inclusion of diverse clinical populations, there are some limitations. First, the study is retrospective and relies on electronic medical records of a tertiary hospital, which may introduce selection bias. Additionally, while AMH is a reliable marker of ovarian reserve, it does not provide a complete picture of fertility potential. Other factors, such as antral follicle count (AFC), FSH levels, and the woman’s overall health, should be considered in conjunction with AMH levels when assessing fertility.

In conclusion, the age-stratified AMH nomogram presented in this study offers a valuable resource for both clinicians and patients. By providing detailed reference ranges across reproductive ages, this nomogram facilitates more accurate assessment of ovarian reserve and empowers women to make informed decisions about their reproductive health. Future research should focus on the longitudinal implications of AMH levels and explore the integration of these nomograms into broader fertility assessment protocols, particularly in populations with specific conditions like endometriosis or polycystic ovary syndrome.

## Data Availability

The raw data supporting the conclusions of this article will be made available by the authors, without undue reservation.
